# Cold atmospheric pressure plasma (CAPP) as a new alternative treatment method for onychomycosis caused by *Trichophyton verrucosum*: in vitro studies

**DOI:** 10.1007/s15010-021-01691-w

**Published:** 2021-09-09

**Authors:** Sebastian Gnat, Dominik Łagowski, Mariusz Dyląg, Jessica Zielinski, Marek Studziński, Aneta Nowakiewicz

**Affiliations:** 1 Department of Veterinary Microbiology, Faculty of Veterinary Medicine, Institute of Preclinical Veterinary Sciences, University of Life Sciences, Akademicka 12, 20-033 Lublin, Poland; 2 Department of Mycology and Genetics, Faculty of Biological Sciences, University of Wroclaw, Wroclaw, Poland; 3 Hollings Cancer Center, Medical University of South Carolina, Charleston, SC USA; 4 Department of Planar Chromatography, Faculty of Chemistry, University of Maria Curie-Sklodowska, Lublin, Poland

**Keywords:** *Trichophyton verrucosum*, Onychomycosis, Nails, CAPP treatment method, Viability

## Abstract

**Purpose:**

Anthropophilic dermatophytes as etiological factors of onychomycoses are more common than zoophilic fungi. In the case of the latter, reverse zoonoses are possible, which poses a threat to the persistence of dermatophytes in the environment. Nevertheless, without treatment, both types of tinea unguium may lead to complete nail plate destruction and secondary mixed infections with fungi and bacteria. One of the zoophilic dermatophytes that cause onychomycosis is *Trichophyton verrucosum*, whose prevalence has been increasing in recent years. Such infections are usually treated with allylamines and/or azoles, but such a conventional treatment of infections caused by *T. verrucosum* often fails or is discontinued by patients.

**Methods:**

Herein, we reveal the results of our in vitro studies related to direct application of cold atmospheric pressure plasma (CAPP) on *Trichophyton verrucosum* growth, germination and adherence to nail as a new alternative treatment method of such types of dermatomycoses.

**Results:**

Our in vitro studies showed that, while exposure to CAPP for 10 min delays germination of conidia and clearly impairs the fitness of the fungal structures, 15 min is enough to kill all fungal elements exposed to plasma. Moreover, the SEM images revealed that *T. verrucosum* cultures exposed to CAPP for 10 and 15 min were not able to invade the nail fragments.

**Conclusion:**

The results revealed that single exposure to CAPP was able to inhibit *T. verrucosum* growth and infection capacity. Hence, cold atmospheric pressure plasma should be considered as a promising alternative treatment of onychomycoses.

## Introduction

Dermatophytoses are superficial infections caused by keratinophilic fungi with high affinity for keratin-rich epidermal layers which can affect the host tissue architecture and functions [1, 2]. Onychomycoses or tinea unguium are a special type of fungal superficial infections of nails representing nearly 50% of all noted onychopathies [3, 4]. These dermatomycoses affect about 10% of the general population with frequencies that vary in different areas of the world and a particularly high percentage in Europe [3]. Factors predisposing to onychomycosis include diabetes, peripheral arterial disease, vascular disease, obesity, vasculopathy, neuropathy, psoriasis, and age, which can contribute to the development of the disease [5, 6]. *Trichophyton rubrum* is the most often isolated etiological factor of nail infections responsible for around 80% cases of all dermatophyte infections in humans [7, 8]. However, non-dermatophyte fungi (NDFs), i.e., *Scopulariopsis brevicaulis*, *Onychocola canadensis*, *Aspergillus* spp., and *Fusarium* spp. can also be etiological factors of this superficial infection [9]. Currently, a great number of reports have revealed increased frequency of yeast dermatomycoses of the nail caused especially by *Candida albicans* [5, 10, 11]. Only few data are available on onychomycoses in humans caused by zoophilic dermatophytes, especially *Trichophyton verrucosum* [12]. This species of dermatophyte is mainly associated with infections in cattle and its breeders, and its prevalence as an etiological factor of onychomycosis has been increasing in recent years [13, 14]. In each case of untreated onychomycosis, complete nail plate destruction and mixed bacterial and fungal secondary infections may develop [4, 5]. In the case of zoophilic onychomycoses, reverse zoonoses are possible [12, 15]. This poses a threat to the persistence of dermatophytes in the environment, taking into account the possibility of an asymptomatic carrier status in animals and close contacts between owners and pets [16].

Superficial mycoses can be treated with systemic or topical antifungals, especially allylamines and/or azoles, depending on the local extension of clinical lesions and pathogen species [2, 4, 5, 17]. However, the long-duration, costs, toxicity, and side effects of the treatment in combination with some other drawbacks of conventional treatment lead to frequent abandonment of therapy or its complete failure in patients and animal owners [18, 19]. Hence, the search for new alternative therapies and antimycotics is of utmost importance.

One of such innovative solutions is the application of plasma medicine, which involves direct application of cold atmospheric pressure plasma (CAPP) on the human or animal body for therapeutic purposes [20, 21]. The antimicrobial effects of cold plasma application have been tested on several groups of microorganisms, e.g., Gram-positive and -negative bacteria, including methicillin-resistant *Staphylococcus aureus* (MRSA) [22]. The antifungal effects of CAPP also showed an in vitro inhibitory effect on *Candida albicans* growth and morphogenesis [23]. The effect of CAPP on dermatophytes has been rarely explored in the literature. Promising data are available for *Trichophyton rubrum* [24]. Moreover, the mechanism of this action is based on suppression of ergosterol biosynthesis in *T. rubrum* after exposure to cold plasma [25]. Thus, the final effect of the action of CAPP is very similar to that observed for azoles and allylamines [26–28].

In the present study, we studied the effect of in vitro CAPP treatment on *Trichophyton verrucosum* isolated as etiological factors of zoonotic onychomycoses in humans. The aim of this study was to determine the viability of dermatophyte cells after different times of exposure to CAPP and to examine its effect on nail plates after in vitro treatment.

## Materials and methods

### Dermatophytes and growth conditions

In total, 12 clinical isolates of *Trichophyton verrucosum* isolated as etiological factors of zoonotic onychomycoses in humans diagnosed from 2012 to 2020 were used in this study. All these isolates showed susceptibility to allylamine-type drugs, including terbinafine and naftifine, and azoles. All clinical isolates were identified to the species level by a combination of conventional and molecular techniques, comprising examination of macro- and micro-morphology and internal-transcribed spacer (ITS) rDNA region sequencing as described previously by Gnat et al. [13]. Re-passage of the isolates was carried out on potato dextrose agar (PDA, Biomaxima, Lublin, Poland) for 7 days at 28 °C. Then, mycelium was gently scrapped by cotton swabs after adding a sterile saline solution (NaCl 0.9%) with 0.002% Tween 20 (Sigma Aldrich, Saint Louis, Missouri, USA). The supernatant containing conidia and mycelium was transferred to a glass tube, and the inoculum was standardized spectrophotometrically at 530 nm to an optical density ranging from 65 to 70% transmission.

### Effect of CAPP on conidia suspension and mycelium

An aliquot of 500 µl of standardized suspension was added to a 24-well titration plate. The suspension was exposed to cold atmospheric pressure plasma (CAPP) for 5, 10, and 15 min. Immediately after the exposure, 500 µl of two times concentrated RPMI broth were added to each well, and then the plates were incubated at 28 °C for 7 days. The growth of exposed and non-exposed fungal strains was measured with a microplate spectrophotometer set (Synergy™ HT, Bio-Tek^®^ Instruments) at 530 nm. The assays were performed in three independent repetitions and each time in triplicate. Throughout the experiment, the conidial germination was observed using an inverted microscope for cell culture (Olympus CX40, magnification 400×). The CAPP device employed in this study was generated as described previously by Borges et al. [23]. Briefly, the 50 cm-long, 2.5-mm-diam polyurethane tube (12 Fr/Ch) and the cooper wire were connected to the primary reactor in which the high-voltage electrode was located. The helium gas was led into the primary reactor. When the discharge was initiated around the electrode, the plasma was excited by the electrode and the plasma stream was generated at the end of the tube. The following parameters were used in this study: the AC voltage signal with a frequency of 32.0 kHz and 13.0 kV, modulated into bursts of ten cycles with a repetition period of 1.5 ms, and 2.0 l/min flow of 99.5% pure helium controlled by a mass flow controller (N100 Horiba STEC, Japan). The distance between the plastic tube and the surface to be treated was set at 1.5 cm. During the plasma treatment, sample temperature was monitored by an infrared thermometer (Daie, model GM-300) and did not exceed 40 °C.

### In vitro infection of human nail

Distal fragments of nail plates trimmed with scissors were collected from volunteers without onychomycoses and any nail disorders. The nails were then cut into 1–2-mm fragments and degreased in 70% ethanol for 15 min. Then, the nails were transferred to 96-well plates containing 50 µl of the dermatophyte cell suspension non-exposed and exposed to CAPP for 5, 10, and 15 min. After 1 h, 150 µl of liquid basal medium (BM) were added to each well. BM was prepared in a final volume of 1 l as previously described by Scott and Untereiner [29]. It contained 15 g of Bacto Agar (Difco, Detroit, MI, USA) and 100 ml of each of the following solutions (prepared individually in a total volume of 1 l): (1) major salt stock solution (5.0 g KCl, 5.0 g MgSO_4_ × 7H_2_O, 0.01 g CaCl_2_ × 2H_2_O); (2) buffer stock solution (14.2 g NaH_2_PO_4_ adjusted to pH 9.0 with a concentrated solution of KH_2_PO_4_); (3) micronutrient stock solution (40 g NaH_2_PO_4_ × H_2_O, 20 g FeCl_3_ × 6H_2_O, and 1 ml each of a solution containing 1000 mg/l MnCl × 6H_2_O, 1000 mg/l ZnSO_4_ × 7H_2_O, 100 mg/l Na_2_MoO_4_ × 2H_2_O, 250 mg/l CuSO_4_ × 5H_2_O). The pH was adjusted to 9.0 by the addition of a solution of concentrated NaOH. The plates were incubated at 28 °C for 10 days. Nail scrapings incubated with the non-exposed and CAPP-exposed dermatophyte cells were fixed with a 2.5% glutaraldehyde solution for 24 h, dehydrated, and analyzed using scanning electron microscopy (SEM, GeminiSEM 360, Zeiss, Germany). Additionally, the suspension of the cells was inoculated onto the SGA (BioMaxima, Lublin, Poland) medium.

### Statistical analysis

The statistical analyses were performed using software Statistica ver. 13.3 (Statsoft, Warsaw, Poland), and the level of significance was set at (*) *p* < 0.05 and (**) *p* < 0.005. The obtained results were averaged and analyzed by normality distribution (Shapiro–Wilk test) followed by ANOVA and Tukey’s post hoc test to compare the intensity of mycelium growth.

## Results

**Fig. 1:**
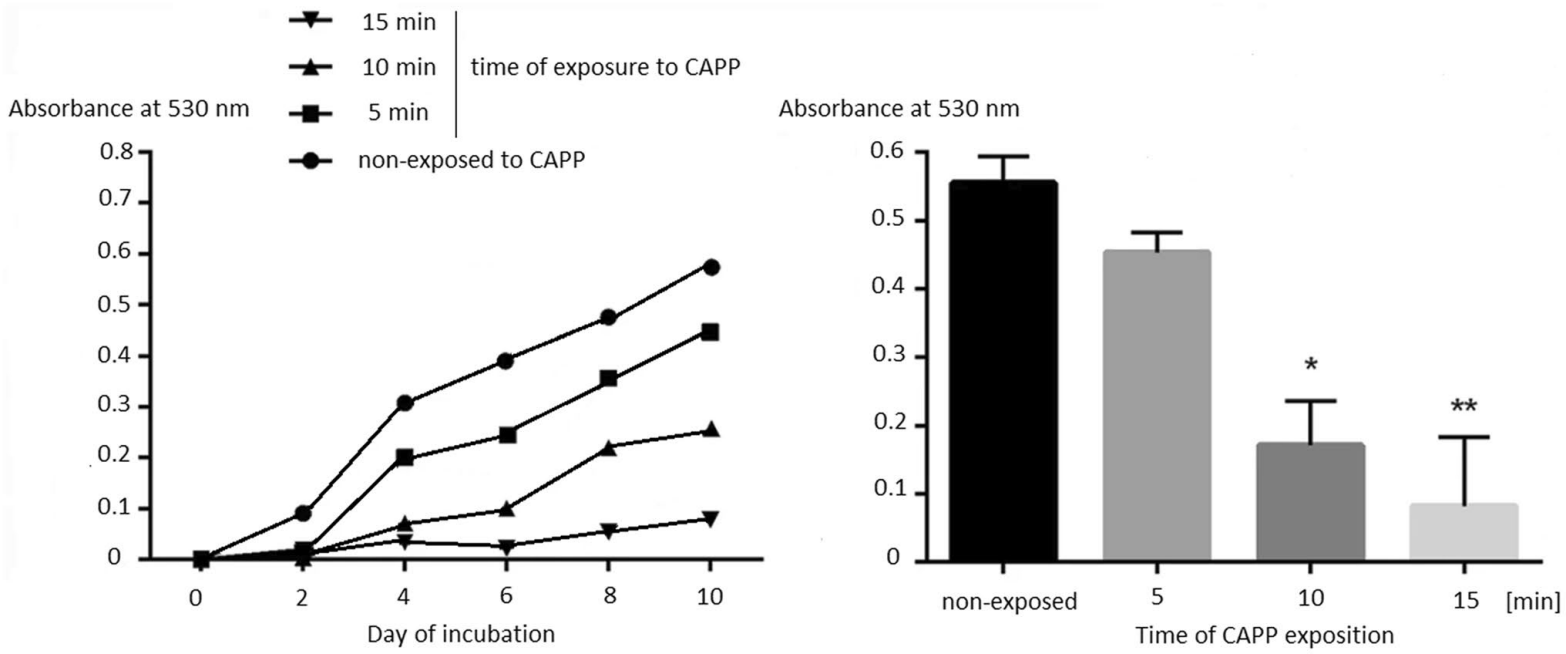
Conidial germination of *Trichophyton verrucosum* isolates obtained from onychomycoses in a 2-day observation cycle and after 10 days exposed to CAPP for 5, 10, and 15 min compared to the non-exposed group based on absorbance measurements at 530 nm (with mean values and standard deviation); (*) *p* < 0.05 and (**) *p* < 0.005

The fungicidal activity of CAPP against conidia and mycelium of *T. verrucosum* after different times of exposure was evaluated spectrophotometrically at 530 nm in 2-day periods up to the 10th day of incubation in optimal conditions (Fig. 1). The activity was reflected in the absence of conidial germination and growth of hyphae in the case of 99.5% of fungal cells. The exposure of the dermatophyte cells to CAPP for 5 min produced the lowest decrease in viability, comparable to the non-exposed suspension of fungal cells, which indicates the weakest antifungal effect. A statistically significant reduction in the viability of fungal cells compared to the CAPP non-exposed cell suspension was demonstrated after 10 (*p* < 0.05) and 15 (*p* < 0.005) minutes of in vitro plasma treatment. The reduction of the total number of viable cells of *T. verrucosum* exposed in vitro to CAPP for 10 and 15 min after 10 days of incubation was three- and sixfold, respectively, compared to the non-exposed cultures. Noticeable differences in the intensity of growth of dermatophytes treated in vitro with plasma for the different exposure times were noted after 4 days of incubation. On the second day of incubation, no difference in the number of germinated conidia and mycelium growth could be detected between groups exposed to CAPP for 5, 10, and 15 min. However, after 2 days of incubation, the non-exposed cultures showed a higher growth rate than those exposed to CAPP. It seems that, regardless of the time of exposure, CAPP reduced *T. verrucosum* growth and germination of conidia, compared to the non-exposed group.

**Fig. 2:**
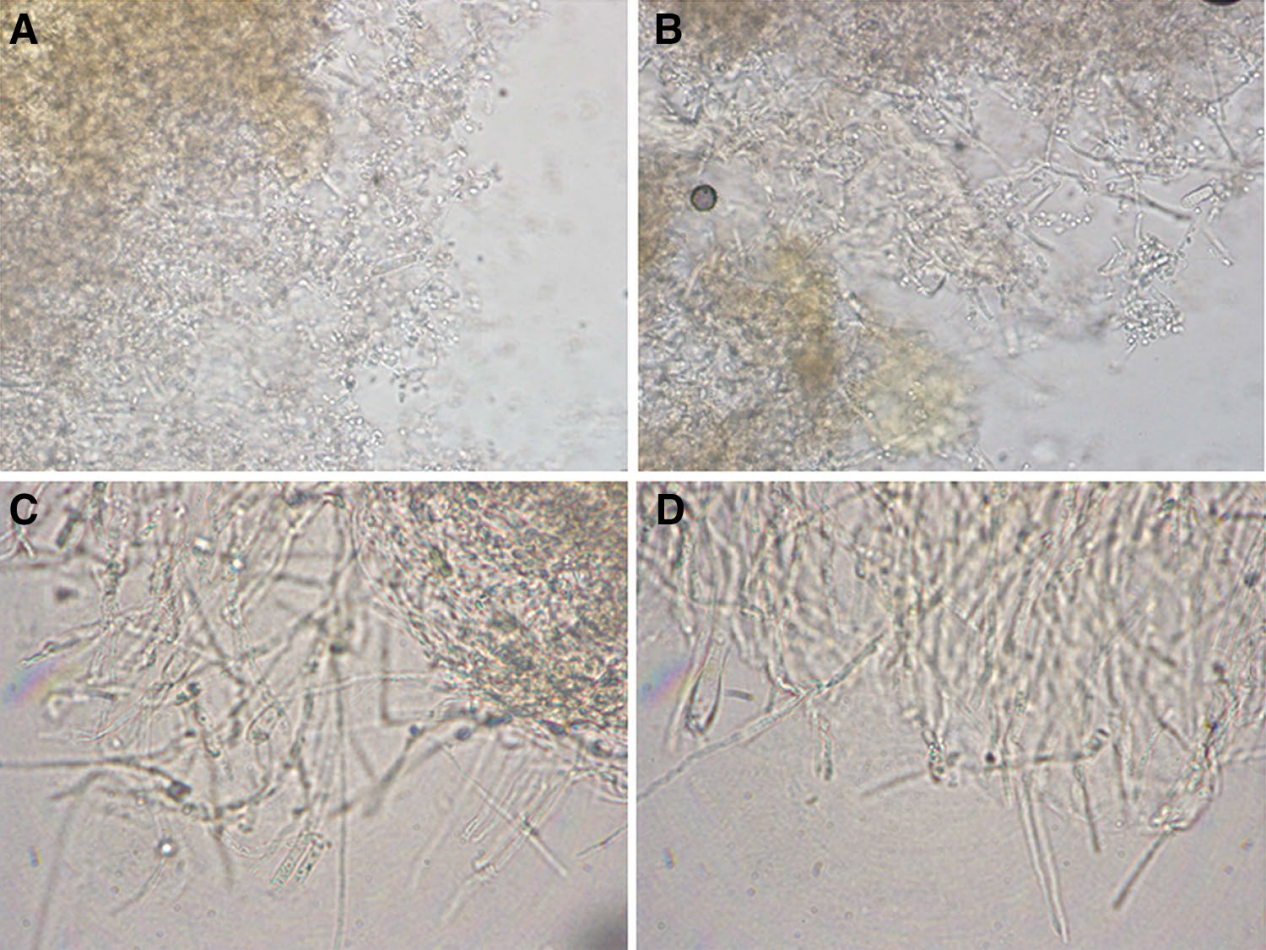
*Trichophyton verrucosum* cell suspensions after 10 days of incubation (microscopic images; Olympus BX51, Tokio, Japan). **A** non-exposed to CAPP; **B** after 5-min exposure to CAPP; **C** after 10-min exposure to CAPP; **D** after 15-min exposure to CAPP

Microscopic slides in physiological saline made from CAPP-exposed and non-exposed dermatophyte cell suspensions after 10 days of incubation are shown in Fig. 2. The examined groups showed distinct transition from the dominant sporous form in the CAPP non-exposed *T. verrucosum* to the hyphal form in cultures exposed to CAPP for 15 min. Conidial germination was observed after 2 days of incubation in the non-exposed groups and after 4 days in the case of cultures exposed to CAPP for 5 min. In the group exposed to CAPP for 10 min, a small quantity of germinated cells was observed only after 10 days. In turn, dermatophyte conidia and hyphae exposed to CAPP for 15 min showed no germination and growth, respectively, even after 10 days of incubation. These results have revealed that CAPP exposure for 10 min delays *T. verrucosum* growth and germination of conidia.

**Fig. 3:**
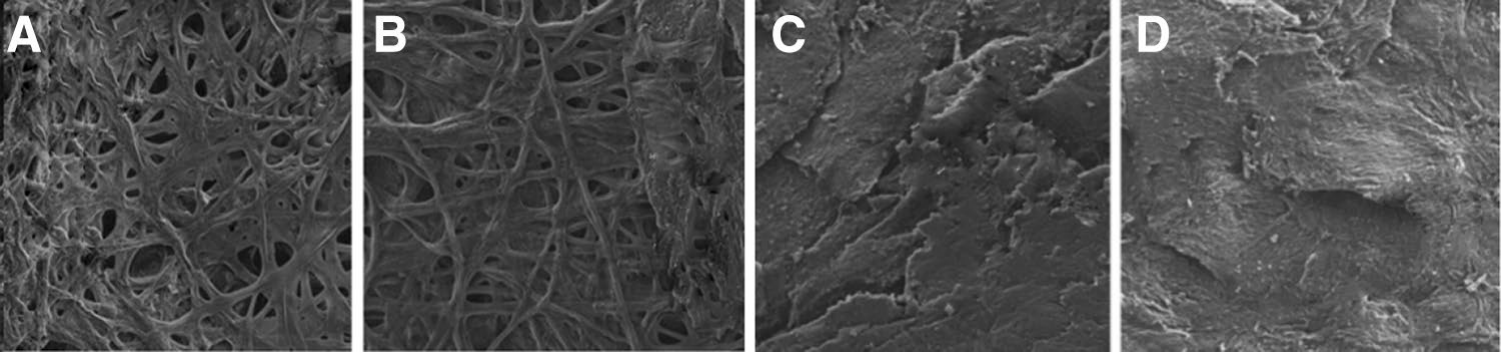
Scanning electron microscopy (SEM) images of nail fragments infected in vitro by *Trichophyton verrucosum* exposed or non-exposed to CAPP. **A** non-exposed to CAPP; **B** after 5-min exposure to CAPP; **C** after 10-min exposure to CAPP; **D** after 15-min exposure to CAPP

To evaluate the ability to invade nail plates by *T. verrucosum* cells, the CAPP-exposed cultures and the non-exposed group were incubated in vitro with human nails. Microscopic examinations using SEM microscopy revealed that the nails incubated with non-exposed and CAPP-exposed dermatophytes for only 5 min were infected after 10 days of incubation. In contrast, *T. verrucosum* cells exposed to CAPP for 10 and 15 min did not grow in vitro in the nails, which proves that the fungi were not able to cause infection (Fig. 3). Additionally, the growth of fungal colonies on SGA medium was observed only in the non-exposed group and that exposed for 5 min. Taken together, these results have revealed that while exposure to CAPP for 10 min delays conidial germination and clearly impairs the fitness of the fungal structures, 15 min is enough to kill all fungal elements exposed to plasma.

## Discussion

Onychomycosis is a common fungal nail infection caused by dermatophytes, mainly *Trichophyton rubrum,* and *T. mentagrophytes*, yeasts, and non-dermatophyte moulds [30]. The prevalence of onychomycosis in the human population has been estimated at values between 10% in Japan and 13.8% in the USA [31]. Available literature data indicate that onychomycoses caused by *T. verrucosum* are very rare, most often being work-related zoonoses [12]. Onychomycoses affect the quality of patient’s life due to the poor appearance of the nail and walking difficulties in the case of toe nail infections [4, 30]. Moreover, untreated infections can be a source of secondary infection or transmission to other family members [32]. In the case of onychomycosis caused by *T. verrucosum* in llama breeders, a case of reverse zoonosis has also been described [12].

In general, oral treatment with itraconazole or terbinafine has been shown to be more effective in onychomycoses with high complete cure rates of 26% and 55%, respectively [33]. However, these therapies have the disadvantages of drug–drug interactions and systemic side effects (e.g., hepatotoxicity) [32]. Furthermore, several antifungals, i.e., itraconazole, terbinafine, amorolfine, and ciclopirox, have been used for topical treatment of onychomycosis based on creams or lacquers [4, 30]. Although topical treatment is not often associated with systemic side effects due to their extremely low capability of penetration from the nail to the bloodstream, complete cure rates are very low, i.e., in the range of 0.96% to approximately 5.5–8.5% [34]. Tachibana et al. [30] revealed that some topical antifungals, i.e., ciclopirox, have high keratin affinity and can bind to keratin in the nail plate losing antifungal potency in keratinized layers. Hence, new strategies for therapy of onychomycoses could improve the efficacy of the treatment.

Cold atmospheric pressure plasma (CAPP) has emerged as a promising microbiologically useful technology in recent years. The antimicrobial effects of CAPP on various vegetative prokaryotic and eukaryotic microorganisms as well as bacterial endospores have been demonstrated in the literature [24, 35–37]. The antimicrobial and antifungal mechanism of action of CAPP is related to production of reactive nitrogen and oxygen species, i.e., ozone, atomic oxygen, superoxide, peroxide, hydroxyl radicals, nitric oxide, and nitrogen dioxide, as well as arousal of energetic electrons and UV radiation, which constitute the plasma air and act synergistically on different biological targets [36–38]. In consequence, the cell membrane integrity is disturbed, and intracellular molecules, such as DNA and proteins, are destroyed resulting in the inhibition of cell growth [37, 39].

The present study has shown that the in vitro CAPP treatment is able to reduce the growth of zoophilic dermatophytes *T. verrucosum* and germination of conidia. The single exposure to CAPP for 10 min completely inhibited conidial germination, sporulation, and significantly inhibited mycelial growth. Moreover, the microscopic appearance of the dermatophyte hyphae was significantly altered morphologically, which indicated their progressive degradation. In contrast, hyphae without any visible morphological changes were observed in the non-exposed group. The 5-min-exposure to CAPP resulted in a 2-day delay in germination, compared to the non-exposed group, and the group exposed to CAPP for 10 and 15 min did not show sporulation and germination at all. These processes can largely impair the progression of the infection. Abnormal and sparse conidia have been found to reduce the infectivity of dermatophytes, and degenerated hyphae penetrate physical barriers in the outer layers of the skin, nails, and hair more weakly [6, 16, 40, 41]. Previous studies with plasma medicine showed that CAPP can be an alternative therapy for onychomycoses and other fungal infections caused by dermatophytes of anthropophilic origin [24, 42, 43]. Similar to our results, Borges et al. [24] revealed that *T. rubrum* radial mycelial growth of germinated and non-germinated conidia was completely inhibited after 10- and 15-min action of CAPP. Contrarily, Heinlin et al. [42] observed that CAPP inhibited *T. rubrum* growth after eight daily exposures to CAPP for 10 min. In their study, a single exposure to CAPP caused only slight inhibition of mycelial growth.

In the context of impaired progression of dermatophytes in onychomycoses, the adherence of conidia and hyphae to nails is considered an important step to infection and a determinant of infectivity [24, 40, 44]. Our results showed that CAPP impaired conidial adherence to nail scrapings. The SEM images revealed that *T. verrucosum* cultures exposed to CAPP for 10 and 15 min were not able to invade the nail fragments. As suggested by Tainwala et al. [40], impaired adhesion is associated with failure of carbohydrate-specific adhesins on the surface of conidia. Our observations show that this may also be caused by significant weakening of sporulation, germination, and damage to vegetative hyphae. The ability to form biofilm is commonly known to play a significant role in the pathogenesis of onychomycosis [5]. Moreover, it is well known that mature biofilms are associated with increased resistance to antifungal therapies [2, 17, 45]. Costa-Orlandi et al. [46] observed that, after 72 h of incubation, dermatophyte cells were embedded within an extracellular polymeric substance, suggesting that the fungi are initiating biofilm formation. The SEM analysis was performed after 10 days of incubation in a mineral medium with nail fragments as a sole carbon source. Hence, CAPP can presumably prevent biofilm formation at the initial step. However, this requires more detailed research.

The in vivo use of CAPP in the treatment of onychomycoses is unknown so far. Here, we present the results of only in vitro studies that provide promising data related to the CAPP antifungal efficacy against dermatophytes. However, since the fungal hyphae in onychomycosis are usually located below the nail plate or in the nail bed, more detailed research is required to determine how deep the therapeutic effect of CAPP in the nail can reach [4]. In addition, it should be taken into account that patients with onychomycosis have mostly thicker nail plates [3, 4]. Current trends in the establishment of CAPP as a robust approach in anti-dermatophyte therapy should also associate with a possible synergic use with commonly used antimycotics. These findings may contribute to extending the application of CAPP to the treatment protocols in antifungal-resistant cases.

In conclusion, CAPP has many promising biomedical applications, such as inactivation of microorganisms [47–49], decontamination of medical equipment [50, 51], blood coagulation [52], wound healing [53, 54], and apoptosis of cancer cells [52]. Depending on the exposure time, the ability to kill fungal cells or inhibit *T. verrucosum* hyphal growth and adhesion to nail plates can be added to this list, as indicated by the results reported herein. Therefore, CAPP should be further investigated as an alternative method of treatment for conventional antimycotics in the therapy of dermatophyte infections.

## Data Availability

The datasets generated during the current study are available from the corresponding author on reasonable request.
